# A TonB-dependent receptor regulates antifungal HSAF biosynthesis in *Lysobacter*

**DOI:** 10.1038/srep26881

**Published:** 2016-05-31

**Authors:** Ruping Wang, Huiyong Xu, Liangcheng Du, Shan-Ho Chou, Hongxia Liu, Youzhou Liu, Fengquan Liu, Guoliang Qian

**Affiliations:** 1 College of Plant Protection (Key Laboratory of Integrated Management of Crop Diseases and Pests), Nanjing Agricultural University, Nanjing 210095, P.R. China; 2 Department of Chemistry, University of Nebraska-Lincoln, Lincoln, Nebraska 68588, United States; 3 Institute of Biochemistry, and NCHU Agricultural Biotechnology Center, National Chung Hsing University, Taichung, Taiwan, ROC; 4 Institute of Plant Protection, Jiangsu Academy of Agricultural Science, Nanjing 210014, P.R. China

## Abstract

*Lysobacter* species are Gram-negative bacteria that are emerging as new sources of antibiotics, including HSAF (Heat Stable Antifungal Factor), which was identified from *L. enzymogenes* with a new mode of action. LesR, a LuxR solo, was recently shown to regulate the HSAF biosynthesis via an unidentified mechanism in *L. enzymogenes* OH11. Here, we used a comparative proteomic approach to identify the LesR targets and found that LesR influenced the expression of 33 proteins belonging to 10 functional groups, with 9 proteins belonging to the TBDR (TonB-Dependent Receptor) family. The fundamental role of bacterial TBDR in nutrient uptake motivates us to explore their potential regulation on HSAF biosynthesis which is also modulated by nutrient condition. Six out of 9 TBDR coding genes were individually in-frame deleted. Phenotypic and gene-expression assays showed that TBDR7, whose level was lower in a strain overexpressing *lesR*, was involved in regulating HSAF yield. TBDR7 was not involved in the growth, but played a vital role in transcribing the key HSAF biosynthetic gene. Taken together, the current *lesR*-based proteomic study provides the first report that TBDR7 plays a key role in regulating antibiotic (HSAF) biosynthesis, a function which has never been found for TBDRs in bacteria.

TonB-dependent receptors (TBDRs) are a family of proteins that are located in the outer membrane of Gram-negative bacteria^1^. These receptors share a common structural feature of two domains; a C-terminal membrane-embedded β-barrel domain that is sealed by a conserved N-terminal globular domain (plug domain)^2^. TBDRs typically act as channels that open in response to outside ligands to allow import of extracellular nutrients, such as iron-siderophore complexes or non-Fe compounds (e.g. vitamin B12), into the periplasmic space^4^. The best-characterized examples include FecA, FhuA, FepA and BtuB, which are necessary for the active transport of the corresponding iron siderophores of ferric citrate, ferrichrome, or enterobactin, as well as vitamin B12, respectively^1^,^2^,^3^. The TBDR-dependent substrate transport is an active process that requires energy input from the proton motive force across the cytoplasmic membrane. Such a process requires that the ligand-loaded TBDRs interact with the TonB protein complex consisting of three inner membrane proteins (TonB/ExbB/ExbD)^5^. Although the basic role of TBDRs is believed mainly in nutrient transport, some TBDRs are also shown to trigger pathogenesis in several animal and plant bacterial pathogens^8^. Nevertheless, TBDR is never reported to play a role in regulating bacterial antibiotic biosynthesis to our knowledge.

LuxR solo is defined as a group of LuxR-family proteins possessing a classical AHL (*N*-acyl-homoserine lactones)-binding domain at the N terminus and a HTH DNA-binding domain at the C terminus, as other LuxR proteins in the canonical LuxI/R system^10^. However, LuxR solo lacks any cognate LuxI protein synthesizing the QS (Quorum sensing) signal AHL^10^,^1^1. Bacterial LuxR solo thus potentially responds to signals produced by the bacteria itself, the neighboring bacteria, or the eukaryotes (e.g. plants) to exert the corresponding regulations, such as biofilm formation, virulence and biocontrol activity in several bacteria^10^,^1^2–1^8^. The genus *Lysobacter*, belonging to the Xanthomonadaceae family, is a group of Gram-negative bacteria with several conserved features, such as high genomic G+C content (approximately 70%), flagella-independent twitching motility, production of abundant lytic enzymes, as well as generation of bioactive natural products^19^,^20^,^21^. These distinct characteristics differentiate *Lysobacter* from its ecological/taxonomic related species, such as *Xanthomonas*^19^,^2^2. A well-characterized species of this genus is *L. enzymogenes*, which is emerging as a biological control agent against fungal pathogens of crop plants, such as *Bipolaris sorokiniana* and *Rhizoctonia solani*^23^,^2^4. *L. enzymogenes* is also currently recognized as a new sources of antibiotics^20^, including HSAF (Heat Stable Antifungal Factor) that belongs to the distinct PTM (polycyclic tetramate macrolactam) antifungal antibiotic with a new mode of action^20^,^2^5,^26^. Furthermore, our previous reports show that the *hsaf pks/nrps* gene, encoding a hybrid polyketide synthase-nonribosomal peptide synthetase, is responsible for the HSAF biosynthesis in *L. enzymogenes*^27^,^2^8. However, the yield of HSAF in *L. enzymogenes* is relatively low even in the HSAF inducing medium^26^,^2^7. Therefore, elucidation of the regulatory mechanism(s) of HSAF biosynthesis in *L. enzymogenes* is necessary for improving the HSAF yield by genetic engineering or molecular biotechnology. Recent advancements have started to shed light into this issue^21^,^2^2,^29^. Intriguingly, we previously found that overexpression, but not deletion of *lesR*, the only LuxR solo coding gene in *L. enzymogenes*, almost entirely impaired the HSAF production^29^. However, how the overexpressed *lesR* performed this critical control on the HSAF biosynthesis remains to be investigated.

To further understand the mechanism used by LesR in regulating HSAF biosynthesis, we have endeavored to identify the LesR targets that contribute to HSAF biosynthesis. By a combination of proteomics, bioinformatics and genetic approaches, we discovered that a certain TBDR protein, whose level was affected by the *lesR* overexpression, played an important role in regulating the antibiotic HSAF biosynthesis in *L. enzymogenes*. Our findings represent the first report about a novel functionality of TBDR proteins in bacteria.

## Results

### The levels of 9 TBDR proteins were affected by the *lesR* overexpression

**Figure 1:**
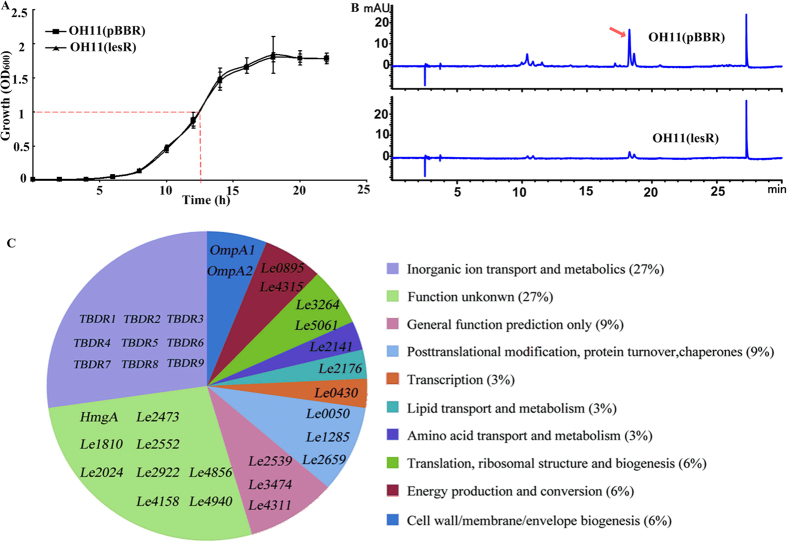
Identification of proteins affected by the *lesR* overexpression through 2-D gel proteome analysis of *Lysobacter enzymogenes* OH11. (**A**) Monitoring and comparison of the growth curve between the *lesR* overexpression and control strains in 1/10 TSB broth. The time point used for cell collection was set at OD_600_1.0 (indicated by the dotted line). Data are from three independent biological experiments. Each experiment involved three replicates for each strain. (**B**) Overexpression of *lesR* almost impaired the HSAF production in strain OH11. The HSAF production (indicated by the red arrow) from the collected cells, as noted in part (**A**), was extracted and determined by HPLC. (**C**) Functional classification of the identified 33 proteins affected by the *lesR* overexpression. The detailed information of each gene described in this figure was provided in the Table 1. OH11(lesR), the *lesR* overexpression strain; OH11(pBBR), the wild-type strain containing an empty expressing vector.

Given that overexpression, but not deletion, of *lesR* in the wild-type OH11 was found to almost shut down the HSAF biosynthesis^29^, we therefore selected the *lesR* overexpression strain, as well as the wild-type OH11 possessing an empty vector as a control for 2-D gel proteome analysis to identify potential LesR targets in *L. enzymogenes*. To achieve this point, the growth ability of the *lesR* overexpression strain and control strain was first determined and compared in the HSAF-inducing medium (1/10 TSB broth). As shown in Fig. 1A, overexpression of *lesR* in the wild-type OH11 did not alter its growth ability in comparison to that of the control strain. On the basis of this result, a good time point at 12.5 h after initial inoculation that corresponds to the logarithmic phase of both strains was chosen for cell collection (Fig. 1A). We showed that regulation of *lesR* in the HSAF biosynthesis was functional at this designated point, because the control strain produced HSAF, whereas no HSAF was detected in the *lesR* overexpression strain at this time (Fig. 1B).

**Figure 2:**
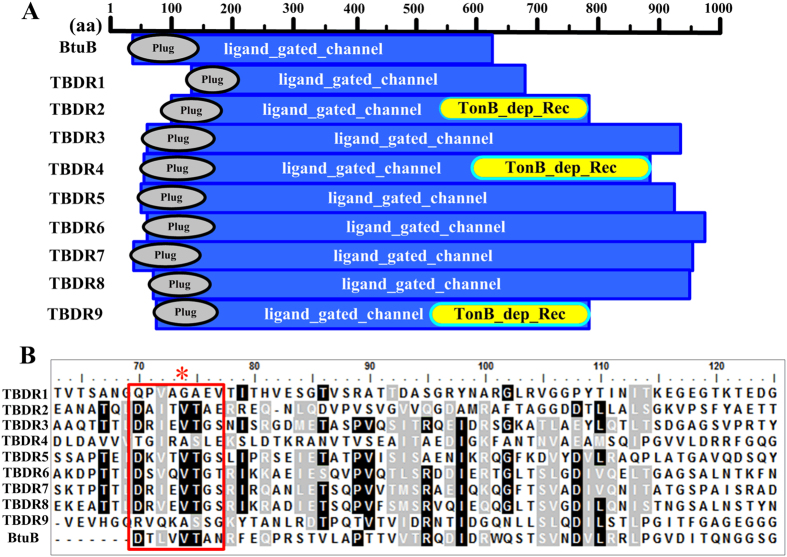
Sequence analysis of nine TBDR proteins identified from the *lesR*-based proteomics in *L. enzymogenes*. (**A**) The domain analyses of nine TBDR (TonB-Dependent Receptor) proteins. The well-characterized vitamin B12 receptor, BtuB from *E. coli* (gi|948468), served as a reference TBDR. (**B**) Multiple alignment of the TonB-box region of TBDR1 to TBDR9 with that of BtuB. The predicted TonB-box region was highlighted with a red box, similar to that of BtuB^30^. The conserved amino acid (Val74) that was marked with a red asterisk was selected for point mutation in further study.

Next, total proteins were extracted from the collected cells of the *lesR* overexpression strain and control strain. After purification and quantification, these proteins were separated by 2-D gel electrophoresis. In this way, a total of 98 differentially expressed protein spots (with a threshold of larger than 1.5-fold change) were excised from silver-stained gels and subject to MALDI-TOF-TOF analysis; 33 of them were confidentially identified in the genome of strain OH11 (Table 1). *In silico* analysis further divided these 33 proteins into 10 groups (Fig. 1C and Table 1). The largest percentage of annotated proteins (27%) affected by *lesR* overexpression corresponds to the group of ‘inorganic ion transport and metabolism’ (Fig. 1C), which comprised 9 TBDR proteins (Table 1). These TBDR proteins were then further investigated for their potential roles in regulating HSAF biosynthesis, because the basic role of TBDR in nutrient uptake in *L. enzymogenes* is speculated to be correlated with the nutrient-dependent property of HSAF biosynthesis^25^,^2^6. After a detailed sequence analysis of these 9 TBDR proteins as shown in Fig. 2A, we found that each TBDR possessed two conserved domains, a C-terminal membrane-embedded β-barrel domain (ligand_gated_channel) and an N-terminal plug domain (Plug domain; ~150–200 residues) that is similar to the well-characterized TBDR protein BtuB^30^,^3^1. Furthermore, TBDR2, TBDR4 and TBDR9 also contained an additional domain, the TonB_dep_Rec domain (TonB dependent_Receptor). Notably, all 9 TBDR proteins had a TonB-box region at their corresponding N terminus as BtuB (Fig. 2B).

The analysis also showed that all detected TBDR proteins did not contain a long N-terminal signaling domain, a distinct structural feature of the TBDT (TonB-dependent transducers) family that differentiates them from the conventional TBDRs^32^. Furthermore, none of all detected TBDR proteins was adjacent to a ECF (extracytoplasmic function) sigma factor and anti-sigma factor in their respective genetic organization (Supplementary Fig. S2), which is another typical characteristic of the TBDT-based CSS (cell-surface signaling) system in bacteria^33^. All these results strongly suggest that the 9 detected TBDR proteins belong to the conventional TBDR family, but not TBDT. Collectively, this 2-D proteomic study indicates that the levels of all 9 TBDR proteins were influenced by the *lesR* overexpression in *L. enzymogenes*.

### Systematic mutation revealed that only TBDR7 played a key positive role in controlling HSAF biosynthesis

**Figure 3:**
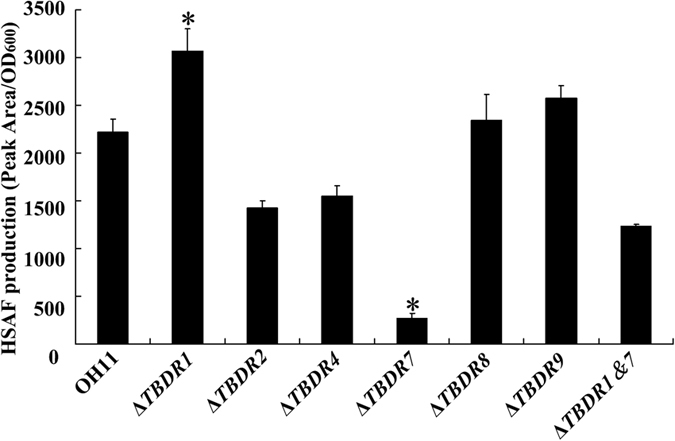
Quantification of HSAF yield from the wild-type OH11 of *L. enzymogenes* and its mutants. Peak area indicated the area of HSAF determined by HPLC method, while the OD_600_ represents the growth status of tested strains at the time points used for the extraction of HSAF. Δ*TBDR* (number) indicated the deletion mutant of target *TBDR* gene; Δ*TBDR1&7*, the double mutant of *TBDR1* and *TBDR7.* Three replicates for each treatment were used, and the experiment was repeated three times. Vertical bars represent standard errors. The asterisk above the bars indicate a significant difference between the wild-type strain OH11 and the tested strains (*p < 0.05).

To test whether all identified TBDR proteins contribute to HSAF biosynthesis, each *TBDR* gene in *L. enzymogenes* was mutated by an in-frame deletion. In this way, 6 gene-deletion mutants, including the Δ*TBDR1, 2, 4, 7, 8* and *9*, were generated and further validated (Supplementary Table S2). The TBDR3, 5 and 6 coding genes appear to be essential for bacterial survival under the test condition, because these gene knockout bacteria failed to grow under a similar condition. Next, the HSAF yield was quantified in each *TBDR* mutant. As shown in Fig. 3, only 2 out of the 6 *TBDR* mutants were found to change the HSAF level, and knockout of the gene *TBDR2*, *4*, *8* or *9* had no effect on the HSAF yield (Fig. 3). In particular, inactivation of *TBDR7* almost abolished the HSAF production, whereas missing of *TBDR1* significantly enhanced the HSAF level (Fig. 3). We also generated a Δ*TBDR1*&*7* double mutant (Table 2 and Supplementary Table S2), and found that it produced approximately 55% HSAF to that of the wild type (Fig. 3), suggesting that TBDR1 and TBDR7 played opposing roles in regulating HSAF biosynthesis in *L. enzymogenes*. Collectively, the above results suggest that both TBDR1 and TBDR7 potentially controlled the HSAF biosynthesis in *L. enzymogenes*.

**Figure 4:**
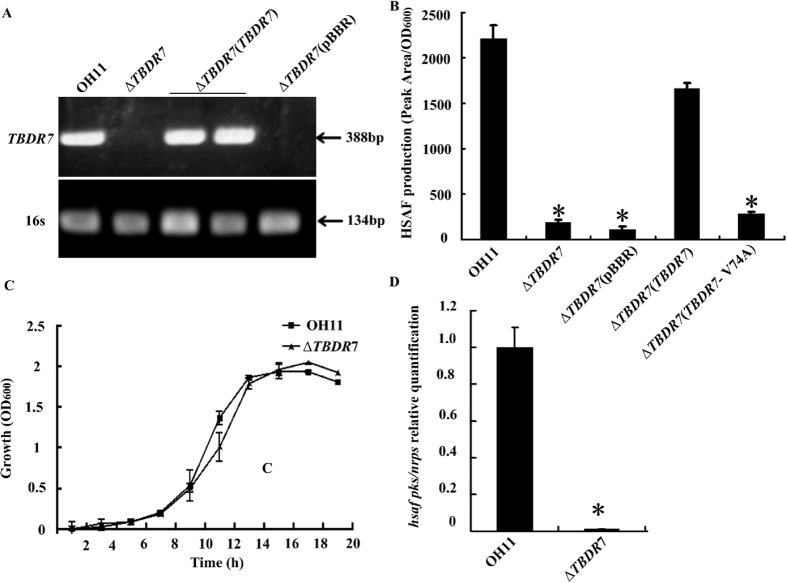
TBDR7 modulated the HSAF production in *L.* enzymogenes. (**A**) RT-PCR assay velidated the transcription of *TBDR7* in the corresponding transformed strain. The size of expected DNA fragment was 388 bp. The gene, *16S rRNA* (abbreviation for *16s*) was used as an internal control, as described previously^22^. (**B**) Quantification of the HSAF level from the complemented strain of the *TBDR7* deletion mutant. (**C**) Growth curves of the wild type and *TBDR7* mutant in 1/10 TSB medium. (**D**) Mutation of *TBDR7* almost entirely shut down the transcription of the key HSAF biosynthetic gene, *hsaf pks/nrps*. OH11, the wild-type strain of *L. enzymogenes*; Δ*TBDR7*, the *TBDR7* mutant; Δ*TBDR7* (pBBR), Δ*TBDR7* containing an empty vector; Δ*TBDR7* (*TBDR7*), the complemented strain of Δ*TBDR7*; Δ*TBDR7* (*TBDR7-*V74A), Δ*TBDR7* containing the plasmid-born *TBDR7*, where the amino acid Val74 within the TonB-box region was substituted by Ala74. Three replicates for each treatment were used, and the experiment was repeated three times. Vertical bars represent standard errors. The asterisk above the bars indicate a significant difference between the wild-type strain OH11 and the tested strains (*p < 0.05).

In the following study, we focused our efforts on TBDR7, because it seems to play a greater role in HSAF production than TBDR1. To verify the role of *TBDR7*, its complemented strain of Δ*TBDR7* was constructed and verified by RT-PCR (Fig. 4A). As shown in Fig. 4B, the *in trans TBDR7* complementation restored the HSAF production of the Δ*TBDR7* mutant to the wild-type level, whereas the Δ*TBDR7* mutant was deficient when complemented with an empty vector (Fig. 4B). In addition, we also created a point mutation (V74A) at the predicted TonB-box region of TBDR7 (Fig. 2B), because this amino acid (Val74) was previously shown to be important in transporting vitamin B12 in BtuB^34^. As shown in Fig. 4B, the TBDR7 containing the V74A indeed failed to complement the HSAF deficiency of the Δ*TBDR7* mutant, revealing the importance of the TBDR7 TonB-box region in controlling HSAF biosynthesis. Collectively, these results suggest that TBDR7 played a vital role in regulating the HSAF production in *L. enzymogenes*.

### TBDR7 positively regulated the *hsaf pks/nprs* transcription

To investigate whether the deficiency of HSAF production of the Δ*TBDR7* mutant in 1/10 TSB is due to the different growth rates, the growth capacity of the Δ*TBDR7* mutant and the wild-type OH11 in this medium was examined. As shown in Fig. 4C, deletion of *TBDR7* did not seem to alter the bacterial growth rate under the similar test condition, suggesting that TBDR7 was not involved in the growth, but controlled the HSAF production in *L. enzymogenes*. To further address this point, we determined the transcriptional level of *hsaf pks/nrps*, the key gene for HSAF biosynthesis in *L. enzymogenes*^27^. As shown in Fig. 4D, transcription of *hsaf pks/nrps* was shut down almost entirely in the Δ*TBDR7* mutant compared to that of the wild-type OH11. This finding was consistent with the decreasing HSAF level in the Δ*TBDR7* mutant (Fig. 3), and further suggests that the contribution of *TBDR7* on the HSAF biosynthesis was, at least partially, due to decreasing transcription of the key HSAF biosynthetic gene in *L. enzymogenes*.

## Discussion

In the present study, an omics-based strategy was utilized to investigate how LesR, the LuxR solo from a biological control agent *L. enzymogenes*, is able to regulate the antibiotic HSAF biosynthesis. A comparative proteomic analysis led to the finding that LesR affects the expression of 98 protein spots when the threshold was set at 1.5-fold change. For these proteins, we have paid attention most to a series of TBDR proteins, because they are closely associated with the nutrient-dependent trait of HSAF biosynthesis. By using a combination of systematic mutation, phenotypic analysis and quantitative gene expression methods, we have further found that TBDR7 was not involved in the growth, and acted as a key protein in controlling the HSAF production. Although bacterial TBDRs have been reported to play a key fundamental role in various nutrient transport, and some of them also have an important role in pathogen-host interaction in several pathogenic bacteria^8^, to our knowledge, no TBDR has been reported to control antibiotic biosynthesis in bacteria^35^. In the present study, we have used *L. enzymogenes* as a model bacterium and provide the first result that TBDR7 was involved in generating HSAF, a unique antifungal antibiotic. The present results therefore reveal a novel function of TBDR in bacteria.

Although the contribution of TBDR7 to the HSAF biosynthesis is well revealed in the present manuscript, the mechanism(s) is still unclear at this moment. It is well accepted that the fundamental function of TBDR is to uptake nutrient in nearly all bacterial species in an energy-dependent way, which requires direct interaction between the periplasmic domain of TonB and the TonB-box region of the ligand-loaded TBDRs^33^. In the present study, we did not yet know the nature of the TBDR7-loading ligand, and also lack the data for the direct interaction between TBDR7 and TonB. However, we did also find that TonB can regulate the HSAF biosynthesis, as mutation of *tonB* (Supplementary Table S2) almost completely impaired the HSAF yield (Supplementary Fig. S3). This result was correlated with the phenotype change of the Δ*TBDR7* mutant on HSAF production. It will be intriguing to identify the potential TBDR7-loading ligand(s) and to explore the possible TBDR7-TonB interaction for a better understanding on how TBDR7 regulates the HSAF biosynthesis.

TBDR7 is one of the 9 TBDR proteins that were identified from a *lesR*-based proteomic study presented in this work. These 9 TBDR proteins account for approximately 16% of all TBDR proteins (55) distributed in the genome of strain OH11 which belong to the group of ‘inorganic ion transport and metabolism’. The functionality of TBDR has been shown to be directly related to different nutrient uptake, such as iron^34^. Competition for iron has long been known to be an important trait for beneficial rhizosphere colonization and for antagonism of plant deleterious microorganisms^36^. This finding has also been reported by PsoR, a LuxR solo of *Pseudomonas fluorescens* that responds to plant compounds^17^. As reported previously, LesR is a LuxR solo, but does not belong to the novel subgroup of plant-responding LuxR solo regulators (e.g. PsoR, OryR and XccR)^29^. However, we found that overexpression of either LesR or PsoR in the background of the relevant wild-type strain affected the gene/proteins involved in iron acquisition^17^ (Fig. 1C). This suggests that different types of LuxR solos from different bacterial biological control agents might share a similar role in controlling expression of the certain genes/proteins, such as those relating to ‘inorganic ion transport and metabolism’.

Since LesR is a transcription factor, one possible mechanism of LesR, therefore, is to regulate transcription of *TBDR7* via a direct or indirect manner. In partial support of this hypothesis, we found that overexpression of *lesR* significantly increased the transcript of *TBDR7* compared to that of the control strain (Supplementary Fig. S4), although the protein level of TBDR7 was decreased in the *lesR* overexpression strain. These results suggest that a post-transcriptional modification may occur in influencing the protein level of TBDR7 in the overexpressed *lesR* strain. We have also attempted to use gel shifting assay to test the direct interaction between LesR and the promoter of *TBDR7 in vitro*, but failed, due to the great difficulty in getting purified recombinant LesR protein. This situation is consistent with the previous report on the preparation of representative LuxR solo OryR from *X*. *oryzae* pv. *Oryzae*^14^,^3^7. In a future study, we will try to fuse the LesR with the MBP tag to obtain soluble fused LesR to check its binding with the *TBDR7* promoter.

## Materials and Methods

### Strains, plasmids and culture conditions

The bacterial strains and plasmids used in this study are listed in Table 2. *Escherichia coli* strain DH5α was used for plasmid constructions, and was grown in LB medium at 37 °C. *Lysobacter enzymogenes* OH11 (CGMCC No. 1978) and its derivative strains were grown in LB or 1/10 TSB (Trypic Soy Broth, Sigma) at 28 °C, shaking at 200 rpm. When required, antibiotics were added into the medium at final concentrations: kanamycin (Km) 100 μg/ml and gentamicin (Gm) 150 μg/ml for *L. enzymogenes* strains; Gm 25 μg/mL for *E. coli* strains.

### Protein extraction and purification, and 2-D gel analyses

Total protein was extracted from bacterial cells according to the previous description^38^. In brief, cells were harvested from the culture (10% TSB broth) with an OD_600_ (Optical Density at 600nm) of 1.0 by centrifugation (10,000 × *g* at 4 °C for 10 min). The cell pellets were re-suspended in 20-mL washing buffer (50 mM Tris-HCI, pH 7.2), and this step (centrifugation and washing) was repeated twice. The final washed cells were resuspended in 400-μl alklysis buffer (addition of 0.1 mM PMSF), and fragmentized by an ultrasonic machine. Then the lysate was discarded by centrifugation at 10,000 × g at 4 C for 20 min. The supernatant was transferred into a fresh 2-mL reaction tube. Total protein of each strain was purified by CKEAN UP kit (GE, USA). Protein samples were subsequently used for 2-D gel analyses, which was described in details in a previous study from our laboratory^39^.

### Trypsin digestion, MALDI-TOF MS analysis and sequence analysis

Protein spots were excised from the 2-D gel. Removal of silver ions in gels, trypsin digestion and peptide extraction were performed as described previously^39^,^4^0. In brief, after the peptides were cocrystallized with CHCA (alpha cyano-4-hydroxy cinnamic acid) by evaporating organic solvents, tryptic-digested peptide masses were measured using a MALDI-TOF-TOF mass spectrometer (ABI4700 System, USA). All mass spectra were recorded in positive reflector mode and generated by accumulating data from 1000 laser shots. The following threshold criteria and settings were used: detected mass range of 700–3200 Da (optimal resolution for the quality of 1500 Da), using a standard peptide mixture [des-Argl-Bradykinin Mr904.468, Angiotensin I Mr1296.685, Glu-l-Fihrinopeptide B Mr1570.677, ACTH (1–17) Mr2093.087, ACTH(18–39) Mr2465.199; ACTH (7–38) Mr3657.929] as an external standard calibration, with laser frequency of 50 Hz, repetition rate of 200 Hz, UV wavelength of 355 nm, and accelerated voltage of 20,000 V. Peptide mass fingerprint data were matched to the comprehensive non-redundant sequence database NCBInr using Profound program under 50 ppm mass tolerance.

Data was processed via the Data Explorer software and proteins were unambiguously identified by searching against the database NCBInr using the MASCOT software search engine (http://www.matrixscience.com/cgi/search form.pl?FORMVER=2&SEARCH=MIS). The search parameters were as follows: (1) peptide quality of 800–4000 Da, mass tolerance for the fragment ion of 0.25 Da; (2) a minimum of seven matching peptides; (3) one missed cleavage; (4) taxonomy: *L. enzymogenes* (bacterium); and (5) allowed modifications, alkylation of cysteine by carbamidomethylation of Cys (complete) acetylation of the N-terminus and oxidation of methionine (partial). Moreover, in order to evaluate protein identification, we considered the percentage of sequence coverage, the observation of distribution of matching peptides (authentic hit is often characterized by peptides that are adjacent to one another in the sequence and that overlap), the distribution of error (distributed around zero), the gap in probability and score distribution from the first to other candidate; only matches with over 90% sequence identity and a maximum *e*-value of 10^−10^ were considered. Domain and functional analyzing of proteins identified was carried out using NCBI (http://www.ncbi.nlm.nih.gov/) and Pfam (http://pfam.sanger.ac.uk/).

### In-frame deletion, complementation and site-directed mutagenesis

The protocol of gene in-frame deletion in *L. enzymogenes* was utilized as described previously^41^. In brief, two flanking regions of target gene were generated by PCR using a set of primer pairs (Supplementary Table S1), and cloned into the corresponding sites of the suicide vector pEX18Gm (Table 2). The final constructs were transformed into the wild-type OH11 or its derivatives by electroporation. Then *Lysobacter* transformants were selected on LB plates without sucrose and with Km (100 μg/ml) and Gm (150 μg/ml). The positive colonies were plated on LB plates containing 1/10 (w/v) sucrose and Km (100 μg/ml) to select for resolution of the construct by a second cross-over event. The resultant mutants were confirmed by a PCR method (Supplementary Table S2). For generation of complemented strains, the target gene, *TBDR7* together with its native promoter region (Supplementary Table S1) was amplified from strain OH11 and cloned into the broad-host vector pBBR1-MCS5 (Table 2). The final construct was transformed into the *TBDR7*-deletion mutant for complementation. The expression of *TBDR7* in the transformed strains was determined by reverse transcription PCR (RT-PCR), which was described as below. Site-directed mutagenesis of the TonB-box region of *TBDR7* was carried out by using the Fast Mutagenesis System kit (FM111-01, Transgen Biotech, China) according to the product manufacturer. The point-mutated DNA was validated by sequencing.

### HSAF extraction and quantification

Extraction and HPLC (High-Performance Liquid Chromatography) based quantification of HSAF from various *Lysobacter* strains was performed as described previously^22^,^2^5,^27^. Three replicates for each treatment were used, and the experiment was performed three times.

### Growth determination

Various *Lysobacter* strains were cultured in LB liquid at 28 °C overnight. Then 500 μl of the overnight culture for each strain was transferred into the fresh 1/10 TSB broth (50 ml) to grow until the OD_600_ reached to 1.0 (the logarithmic phase of growth). Next, 1 ml of each culture was transferred again into the fresh 1/10 TSB broth (50 ml) to start the detection of growth curve. All inoculation broths were grown at 28 °C with shaking at 200 rpm, and the OD_600_ value was determined every 2 h until bacterial growth reached the stationary phase. Each sample involves three technical replicates and the experiment was performed three times.

### RNA extraction, qRT-PCR and RT-PCR

The wild-type OH11 and its derivatives, including the *TBDR*7 deletion mutant, OH11 containing an empty vector and the *lesR* overexpression strain, were grown on 1/10 TSB. Cells of the wild-type OH11 and the *TBDR*7 mutant were collected at the time point 10 h and 11 h, respectively, corresponding to the same cell density (OD_600_ = 1.0). Similarly, OH11 containing the empty vector and the *lesR* overexpression strain were collected at the time point 12.5 h (OD_600_ = 1.0). Then the total RNA was extracted from the collected cells of each strain using a kit from OMIGA Company (China). Next, qRT**-**PCR (quantitative RT**-**PCR) and RT-PCR assays, including cDNA synthesis and PCR amplification were performed as described previously^22^,^2^9. Primer sequences used in this assay were listed in Supplementary Table S1.

### Data submission

The sequence data of the present study has been submitted to the NCBI Genbank. The details were provided in Table 1.

### Data analysis

All analyses were conducted using SPSS 14.0 (SPSS Inc., Chicago, IL, USA). The hypothesis test of percentages (*t*-test, *p < 0.05) was used to determine significant differences in production of antibiotic metabolite and gene expression between the control and treatment of the present study.

## Additional Information

**How to cite this article**: Wang, R. *et al.* A TonB-dependent receptor regulates antifungal HSAF biosynthesis in *Lysobacter*. *Sci. Rep.* **6**, 26881; doi: 10.1038/srep26881 (2016).

## Supplementary Material

Supplementary Information

## Figures and Tables

**Table 1:** Identification of 33 proteins affected by the *lesR* overexpression in *Lysobacter enzymogenes*.

Catalog no.	Spot no.	Fold change OH11(PBBR)/OH11(*lesR*)	Sequence coverage (%)	Gene accession no.	Gene name^a^/abbreviation	Functional catalog^b^	Function/Similarity	pl (cal)	*M*_r_ (cal) KD
1	7-03	+1000000	22	KP293905	TBDR9	Inorganic ion transport and metabolis	TonB-dependent siderophore receptor	5.26	83.5
2	7-05	+3.71567	10	KP293900	TBDR4		TonB-dependent receptor	4.95	94.9
3	7-12	−2.42089	23	KP293903	TBDR7		TonB-dependent outer membrane receptor	4.86	103.9
	7-13	−1000000	15						
	7-15	−1000000	12						
	7-16	−2.95065	9						
	7-18	−1000000	19						
	7-30	−1000000	13						
4	7-19	+2.36877	18	KP293903	TBDR8		TonB-dependent receptor	4.89	102.7
	7-28	−6.99916	21						
5	7-20	+6.49466	15	KP293902	TBDR6		TonB-dependent receptor	5.06	105.5
	7-21	−6.21763	10						
6	7-38	+3.28287	9	KP293901	TBDR5		TonB-dependent receptor	5.24	102.3
7	8-03	−1000000	24	KP293901	TBDR3		TonB-dependent receptor	5.39	99.0
8	8-06	−2.06331	27	KP293898	TBDR2		TonB-dependent receptor domain protein	5.60	86.2
9	7-49	+1000000	6	KP293897	TBDR1		putative tonb-dependent outer membrane receptor	5.64	118.6
10	7-07	+1000000	28	KP293926	OH11GL004315/le4315	Energy production and conversion	Dihydrolipoyl dehydrogenase	6.03	50.4
11	8-22	−2.28895	30	KP293925	OH11GL000895/le0895		catalytic domain of components of various dehydrogenase complexes	6.32	46.5
12	7-14	+3.75737	18	KP293921	OH11GL002176/le2176	Lipid transport and metabolism	FadL family outer membrane protein	5.27	48.4
	7-42	−1000000	26						
13	7-25	+1000000	22	KP293928	OmpA2	Cell wall/membrane/envelope biogenesis	OmpA family outer membrane protein	4.8	39.1
14	7-34	+1000000	28	KP293927	OmpA1		OmpA family outer membrane protein	4.8	39.1
15	7-26	+2.82758	19	KP293917	OH11GL000050/le0050	Posttranslational modification, protein turnover, chaperones	trigger factor	4.96	48.8
	7-45	−1000000	16						
	7-51	−8.98225	8						
16	7-33	+1000000	38	KP293918	OH11GL001285/le1285		glutaredoxin-like protein	4.98	32.7
	7-46	−3.51678	44						
17	8-44	−2.28179	13	KP293918	OH11GL002659/le2659		chaperonin GroEL	5.2	57.3
18	7-22	−1000000	22	KP293907	OH11GL001810/le1810	Function unknown	DNA binding domain-containing protein	4.85	38.1
19	7-23	+1000000	53	KP293910	OH11GL002922/le2922		hypothetical protein	5.73	35.1
20	7-28	+6.99916	21	KP293912	OH11GL004158/le4158		No hit	5.22	86.7
21	7-39	−2.05056	58	KP293908	OH11GL002552/le2552		hypothetical protein	5.34	29.8
22	7-43	+1000000	17	KP293913	OH11GL004940/le4940		No hit	8.35	26.6
23	7-44	+1000000	24	KP293906	OH11GL002473/le2473		No hit	5.15	40.8
24	8-30	−2.4328	34	gi|189474077	*hmgA*		homogentisate 1, 2-dioxygenase	5.93	50.1
25	8-38	+2.00608	40	KP293909	OH11GL002024/le2024		putative secreted protein	6.77	31.8
26	8-41	+2.54476	39	KP293911	OH11GL004856/le4856		No hit	5.2	26.6
27	7-32	+3.73628	17	KP293915	OH11GL003474/le3474	General function prediction only	hypothetical protein	4.69	24.4
28	7-35	+1000000	29	KP293916	OH11GL004311/le4311		hypothetical protein	5.78	34.2
29	8-35	−2.79579	47	KP293914	OH11GL002539/le2539		NADP-dependent alcohol dehydrogenase	5.43	38.06
30	7-36	+2.63927	38	KP293922	OH11GL002141/le2141	Amino acid transport and metabolism	spermidine synthase	5.05	31.8
31	8-02	+3.82444	18	KP293920	OH11GL000430/le0430	Transcription	DNA-directed RNA polymerase subunit beta	5.73	155.2
32	8-34	−2.02788	33	KP293920	OH11GL003264/le3264	Translation, ribosomal structure and biogenesis	glutamyl-tRNA synthetase	5.56	51.6
33	8-47	+1000000	18	KP293924	OH11GL005061/le5061		No hit	5.4	43.2

^a^Gene name was based on the genome sequence of *L. enzymogenes* strain OH11^27^, which could be found with the accession number 1784099 in NCBI database.

^b^Functional catalog was performed by using protein blast (http://blast.ncbi.nlm.nih.gov/Blast.cgi).

**Table 2:** Bacterial strains and plasmids used in this study.

Strains and plasmids	Properties or characteristics^a^	Source
*Lysobacter enzymogenes*		
OH11	The wild-type strain, Km^R^	CGMCC No. 1978
OH11(pBBR)	OH11 harboring plasmid pBBR1-MCS5, Gm^R^, Km^R^	29
OH11(*lesR*)	OH11 harboring plasmid pBBR-*lesR*, Gm^R^, Km^R^	29
Δ*TBDR1*	*TBDR1* in-frame deletion mutant of strain OH11, Km^R^	This study
Δ*TBDR2*	*TBDR2* in-frame deletion mutant of strain OH11, Km^R^	This study
Δ*TBDR4*	*TBDR4* in-frame deletion mutant of strain OH11, Km^R^	This study
Δ*TBDR7*	*TBDR7* in-frame deletion mutant of strain OH11, Km^R^	This study
Δ*TBDR8*	*TBDR8* in-frame deletion mutant of strain OH11, Km^R^	This study
Δ*TBDR9*	*TBDR9* in-frame deletion mutant of strain OH11, Km^R^	This study
Δ*TBDR1&7*	A double in-frame mutation in *TBDR1* and *TBDR*, Km^R^	This study
Δ*tonB*	*tonB* in-frame deletion mutant of strain OH11, Km^R^	This study
Δ*TBDR7*(*TBDR7*)	Δ*TBDR7* harboring plasmid pBBR-*TBDR7*, Gm^R^, Km^R^	This study
Δ*TBDR7*(pBBR)	Δ*TBDR7* harboring plasmid pBBR-MCS5, Gm^R^, Km^R^	This study
Δ*TBDR7*(*TBDR7*-V74A)	Δ*TBDR7* harboring plasmid pBBR-*TBDR7*, Gm^R^, Km^R^	This study
*Escherichia coli*		
DH5a	F^−^, φ80d*lacZ*∆M15, ∆(*lacZYA*-*argF*)U169, *deoR*, *recA1*, *endA1*, *hsdR*17(r_k_^−^,m_k_^+^), *phoA*, *supE*44, λ^−^, *thi*-1, *gyrA*96	Lab collection
Plasmids		
pEX18GM	Suicide vector with a *sacB* gene, Gm^R^	42
pBBR1-MCS5	Broad-host-range vector with a P_*lac*_ promoter, Gm^R^	43
pBBR-*lesR*	pBBR1-MCS5 cloned with a 1525-bp fragment containing the coding region of *lesR* and its predicted promoter region	29
pEX18-*TBDR*1	Km^R^, pEX18GM with two *TBDR*1 flanking fragments	This study
pEX18-*TBDR*2	Km^R^, pEX18GMwith two *TBDR*2 flanking fragments	This study
pEX18-*TBDR*4	Km^R^, pEX18GM with two *TBDR*4 flanking fragments	This study
pEX18-*TBDR*7	Km^R^, pEX18GM with two *TBDR*7 flanking fragments	This study
pEX18-*TBDR*8	Km^R^, pEX18GM with two*TBDR*8 flanking fragments	This study
pEX18-*TBDR*9	Km^R^, pEX18GM with two*TBDR*9 flanking fragments	This study
pEX18-*tonB*	Km^R^, pEX18GM with two*TonB* flanking fragments	This study
pBBR-*TBDR*7	pBBR1-MCS5 cloned with a 3266-bp fragment containing the coding region of *TBDR7* and its predicted promoter	This study
pBBR-*TBDR*7(V74A)	pBBR-*TBDR*7, where the amino acid V74 within the TonB-box region was substituted by A74	This study

^a^Km^R^ and Gm^R^ = Kanamycin-, Gentamicin-, respectively.
