# Supplementary material for: A TonB-dependent receptor regulates antifungal HSAF biosynthesis in Lysobacter
Source: Sci Rep. 2016 May 31;6:26881. doi: 10.1038/srep26881 (PMC4886534; doi:10.1038/srep26881)
Supplement: Supplementary Information [file srep26881-s1.pdf]

Supplementary Materials

A TonB-dependent receptor regulates antifungal HSAF biosynthesis in *Lysobacter*

Ruping Wang[a], Huiyong Xu[a], Liangcheng Du[c], Shan-Ho Chou[d], Hongxia Liu[a], Youzhou Liu[b], Fengquan Liu*[b] and Guoliang Qian*[a]

[a] College of Plant Protection, Nanjing Agricultural University, Nanjing 210095, China/Key Laboratory of Integrated Management of Crop Diseases and Pests (Nanjing Agricultural University), Ministry of Education

[b] Institute of Plant Protection, Jiangsu Academy of Agricultural Science, Nanjing 210014, P.R. China

[c] Department of Chemistry, University of Nebraska-Lincoln, Lincoln, Nebraska 68588, United States

[d] Institute of Biochemistry, and NCHU Agricultural Biotechnology Center, National Chung Hsing University, Taichung, Taiwan, ROC

*To whom correspondence should be addressed. Tel: +86-25-84396109. Fax: +86-25-84396109. E-mail: glqian@njau.edu.cn **or** fqliu20011@sina.com

Outlines

Figure S1. 2-D gel proteome analysis of the *lesR* overexpression strain.

Figure S2. The genomic organization of each *lesR*-controlled TBDR coding genes in *L. enzymogenes*.

Figure S3. Quantification of the HSAF yield from the *tonB* mutant of *L. enzymogenes*.

Figure S4. Overexpression of *lesR* caused an approximately twice increase on the transcription of *TBDR7* in comparison to the control strain.

Table S1. Primers used in this study

Table S2. Mutant confirmation by PCR in this study

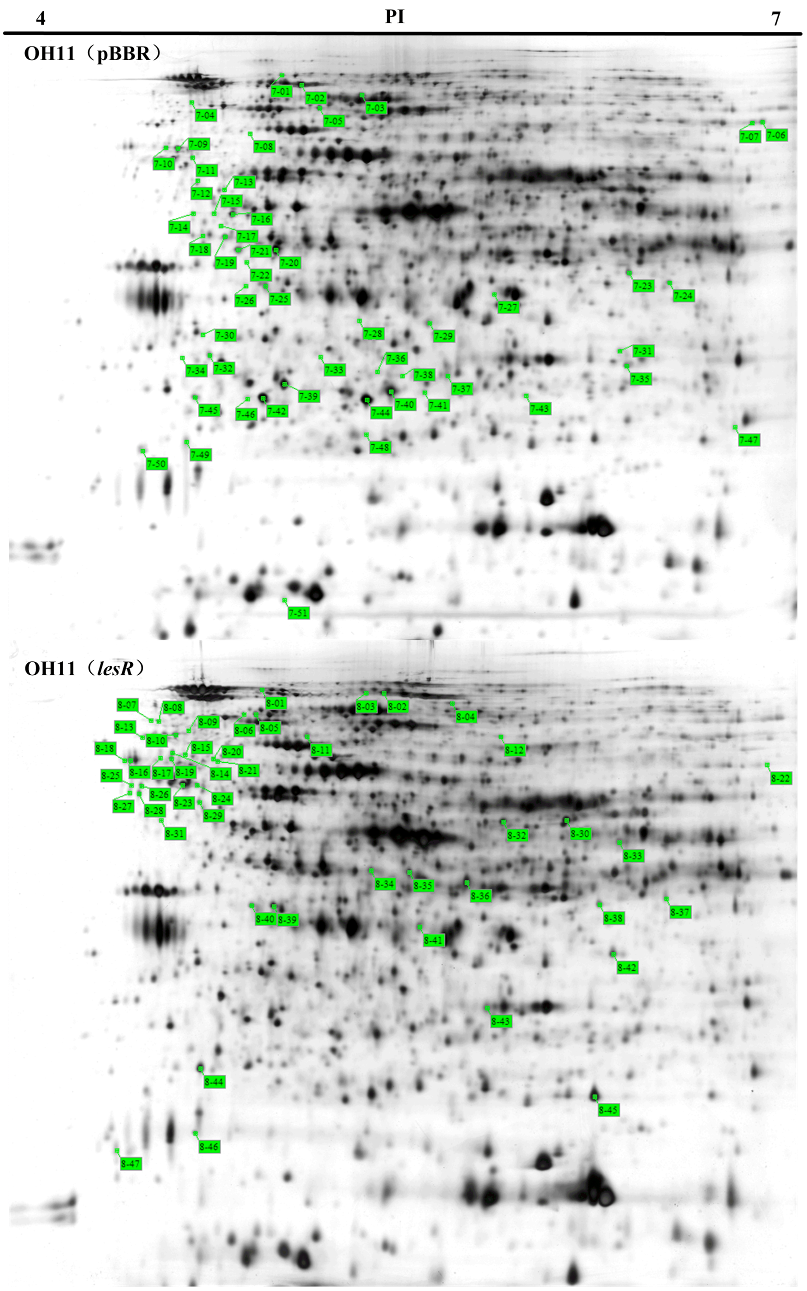

Figure S1. 2-D gel proteome analysis of the *lesR* overexpression strain. The up-expressed protein spots with the threshold ratio of 1.5 were numbered in green either in OH11 (pBBR) or OH11 (*lesR*) compared to each other. Detailed information of the identified *lesR*-controlled proteins was provided **Table 2**. OH11(pBBR), the control strain, representing the wild-type OH11 of *L. enzymogenes* containing an empty expressing vector; OH11(*lesR*), the *lesR* overexpression strain.

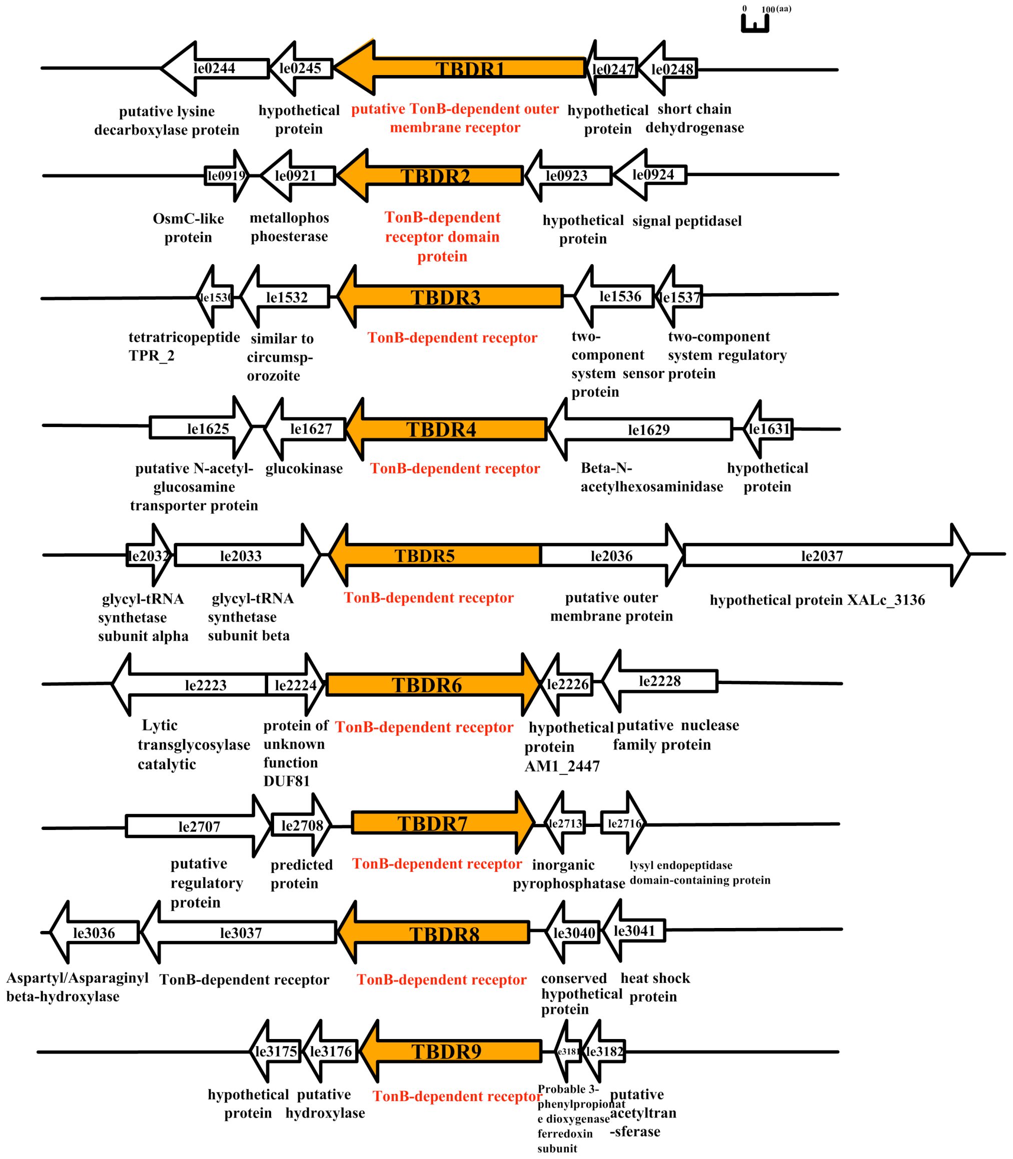

Figure S2. The genomic organization of each *lesR*-controlled TBDR coding genes in *L. enzymogenes*. Each TBDR was colored, whereas other genes were shown in white arrows. The predicted gene products were provided below each arrow. The scale indicates the amino-acid size was identical to 100 aa.

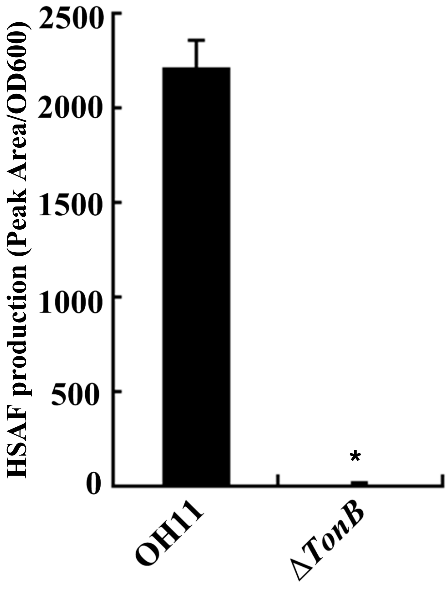

Figure S3. Quantification of the HSAF yield from the *tonB* mutant of *L. enzymogenes*. Mutation of *tonB* (GU121672) almost completely abolished the HSAF production. Three replicates for each treatment were used, and the experiment was repeated three times. Vertical bars represent standard errors. The asterisk above the bars indicate a significant difference between the wild-type strain OH11 and the tested strain (*$p < 0.05$).

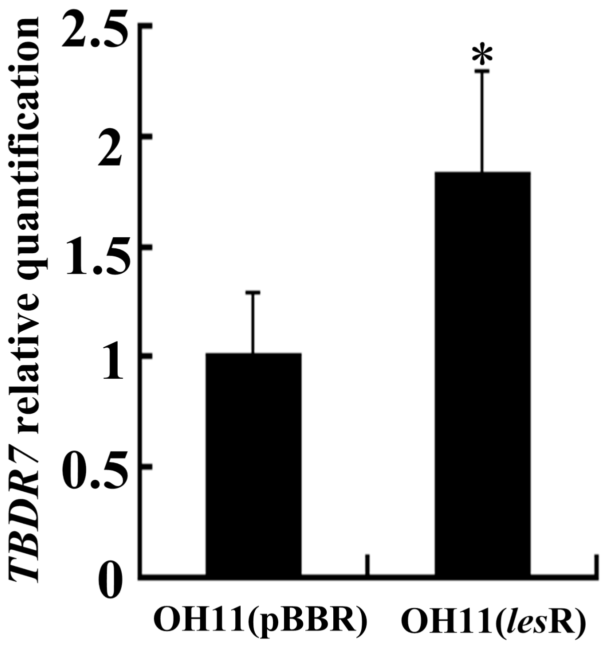

Figure S4. Overexpression of *lesR* caused an approximately twice increase on the transcription of *TBDR7* in comparison to the control strain. The control strain is OH11 (pBBR), representing the wide-type OH11 containing an empty vector; OH11 (*lesR*), the *lesR* overexpressing strain. Each column indicates the mean of three biologically independent quantitative RT-PCR experiments. Vertical bars represent standard errors. The asterisk above the bars indicate a significant difference between the wild-type strain OH11 and the tested strains (*$p < 0.05$).

Supplementary reference

1. Qian, G. L. *et al. Lysobacter enzymogenes* uses two distinct cell-cell signaling systems for differential regulation of secondary-metabolite biosynthesis and colony morphology. *Appl. Environ. Microbiol.* **79**, 6604-6616 (2013a).
2. Schöffler, H. & Braun, V. Transport across the outer membrane of *Escherichia coli* K12 via the FhuA receptor is regulated by the TonB protein of the cytoplasmic membrane. *Mol. Gen. Genet.* **217**, 378-383 (1989).

Table S1 Primers used in this study

Primer	Sequence [a]	Purpose	Source
TBDR1-1F	5'-GGGGTACCCGACTCCGCCACGCATTCCA-3'	To amplify a 314-bp upstream homologue arm of *TBDR1*	This study
TBDR1-1R	5'-CCCAAGCTTTCGCCTTCCTTGGTGATGTTGA-3'		
TBDR1-2F	5'-CCCAAGCTT CCGGCTGGAACTACAACTACCT-3'	To amplify a 1054-bp downstream homologue arm of *TBDR1*	This study
TBDR1-2R	5'-GCTCTAGA CGGCGGAAATCACGAAAA-3'		
TBDR2-1F	5'-CCCGGTACCGAGGGCGGTGCGGAGTTC-3'	To amplify a 788-bp upstream homologue arm of *TBDR2*	This study
TBDR2-1R	5'-CCCTCTAGACAGCAGCAGGCTGGCGAC-3'		
TBDR2-2F	5'-CCCTCTAGACGCAACATCACCAACGAGAAGA-3'	To amplify a 999-bp downstream homologue arm of *TBDR2*	This study
TBDR2-2R	5'-CGGAATTCAGCGAGTACAGCAGCCACAGC-3'		
TBDR4-1F	5'-CGGAATTCGGTGCCAGTGGAAGGTGTTGAG-3'	To amplify a 1062-bp upstream homologue arm of *TBDR4*	This study
TBDR4-1R	5'-CCCAAGCTTAGCGTGGCCTGGAACATCG-3'		
TBDR4-2F	5'-CCCAAGCTTCGTTGGTGTTGGCGAACTTGC-3'	To amplify a 985-bp downstream homologue arm of *TBDR4*	This study
TBDR4-2R	5'-GCTCTAGAGCGGACATGCTGCGTTTGGT -3'		
TBDR7-1F	5'-GGGGTACCCGGCTACTCGCACATCCACG-3'	To amplify a 503-bp upstream homologue arm of *TBDR7*	This study
TBDR7-1R	5'-GCTCTAGACGCCAACACCTTCCACGACG-3'		
TBDR7-2F	5'-GCTCTAGACGGTGGCGGTGATGTTCTGG-3'	To amplify a 554-bp downstream homologue arm of *TBDR7*	This study
TBDR7-2R	5'-CGGGATCCAACGCACGAACCCGCATCCG-3'		
TBDR8-1F	5'-GGGGTACCAACGGCGACAAGGACGAAGG-3'	To amplify a 806-bp upstream homologue arm of *TBDR8*	This study
TBDR8-1R	5'-CCCAAGCTTCGTTGCCGCCGTTGTTGTAG-3'		
TBDR8-2F	5'-CCCAAGCTTGGCAACTGGTCGGAGGGCTT-3'	To amplify a 681-bp downstream homologue arm of *TBDR8*	This study
TBDR8-2R	5'-GCTCTAGAGACCGAGCCCAGTTCCCAGT-3'		
TBDR9-1F	5'-GGGGTACCAGTTCGTCGTGTCGCCGCTC-3'	To amplify a 739-bp upstream homologue arm of *TBDR9*	This study
TBDR9-1R	5'CCCAAGCTTGGGGTGTTGTCGTCGCTCTG-3'		
TBDR9-2F	5'-CCCAAGCTTGCAAGAACTACAAGGTCGCC-3'	To amplify a 1052-bp downstream homologue arm of *TBDR9*	This study
TBDR9-2R	5'-GCTCTAGATTCGCCACCGTCGTATTCGT-3'		
tonB-1F	5'-ACGAATTCAACACCAGCGAGCAGTTGTT-3'	To amplify a 706-bp downstream homologue arm of *tonB*	This study
tonB-1R	5'-CCAAGCTTGTGCTGTTCGGCTTCAATTC-3'		
tonB-2F	5'-AAAAGCTTGCTGCCGCACAGCGTGTCGA-3'	To amplify a 738-bp downstream homologue arm of	This study

tonB-2R	5'-AATCTAGAATGCCGCCCCTGGCGATCCG-3'	*tonB*	
tonB-F	5’-GATTTCGTCCCGCCGAACTG -3’	Mutant confirmation: to amplify a 991- and 250-bp DNA band from the wild-type OH11 and *tonB*-deletion mutant, respectively	This study
tonB-R	5’-TGATCAGCAGCACCAGCATC-3’		
TBDR7-F	5’-GCTCTAGAGGTGTCGGCACTGGTCCCGGCGAT-3’	To amplify 3266-bp fragment of *TBDR7* with its predicted promoter	This study
TBDR7-R	5’-GGGGTACCTCAGAAGCGCTGGGTGTACTTC-3’		
TBDR7-Fm	5'-TCTCGATCGCATCGAGGCGACCGGTTCGCG-3'	To amplify pBBR-*TBDR7*(site directed mutagenesis), the box indicated the target point mutation (V74 was changed into A74)	This study
TBDR7-Rm	5'-GCCTCGATGCGATCGAGAGTGGTGGGGGTC-3'		
16S rDNA-1	5'-ACGGTCGCAAGACTGAAACT-3'	The internal control for RT-PCR and quantitative RT-PCR	1
16S rDNA-2	5'-AAGGCACCAATCCATCTCTG-3'		
q *pks/nrps*-F	5'-CATCACATCATCTCCGATGC-3'	To determine the transcriptional level of the gene *pks/nrps*	1
q *pks/nrps*-R	5'-CAGTTCCACCTTCTCCTTGC-3'		
q*TBDR7*-F	5'-ACAACCAGAACAAGGGCAAC-3'	To determine the transcriptional level of the gene *TBDR7*	This study
q*TBDR7*-R	5'-GCTGAGGTTGAGGCTGTAGA-3'		

[a] Restricted digestion enzyme site was underlined.

Table S2 Mutant confirmation by PCR in this study

Mutant	Selected primers [a]	Expected size from wild type	Expected size from mutant	PCR validation [b]
Δ*TBDR1*	*TBDR1*-1F/2R	3629 bp	1368 bp	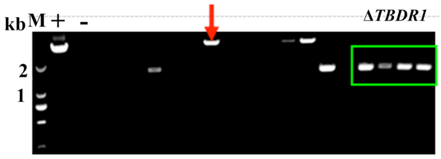
Δ*TBDR2*	*TBDR2*-1F/2R	3944 bp	1787 bp	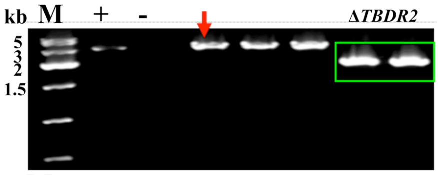
Δ*TBDR4*	*TBDR4*-1F/2R	4267 bp	2047 bp	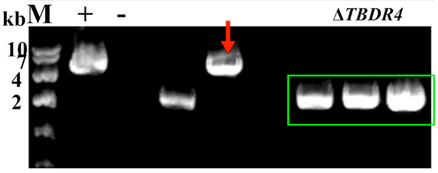
Δ*TBDR7*	*TBDR7*-1F/2R	2604 bp	1057 bp	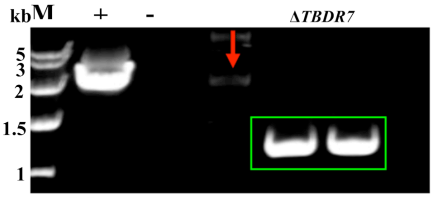
Δ*TBDR8*	*TBDR8*-1F/2R	3016 bp	1487bp	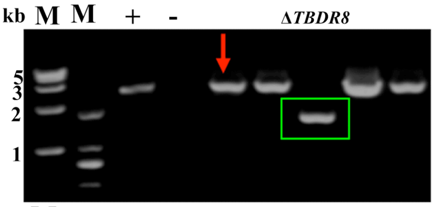
Δ*TBDR9*	*TBDR9*-1F/2R	2789 bp	1791 bp	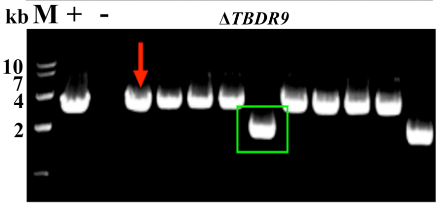
Δ*TBDR1&7*	*TBDR1*-1F/2R	3629 bp	1368 bp	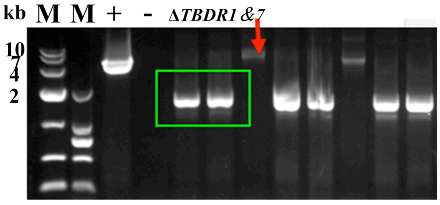
tonB	*tonB*-F/R	991bp	250bp	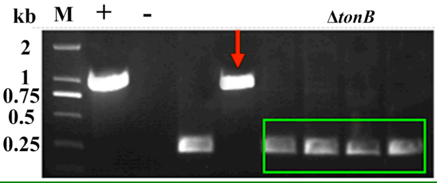

[a] Primers sequence used here were provided in Supplementary Table S1.
[b] Red arrow and green box indicated the expected size from the wild-type OH11 or deletion mutant amplified by selected primers, respectively; + and – means the positive control (the genomic DNA of strain OH11) and negative control (ddH_2O), respectively.
